# Avian Cone Photoreceptors Tile the Retina as Five Independent, Self-Organizing Mosaics

**DOI:** 10.1371/journal.pone.0008992

**Published:** 2010-02-01

**Authors:** Yoseph A. Kram, Stephanie Mantey, Joseph C. Corbo

**Affiliations:** Department of Pathology and Immunology, Washington University School of Medicine, St. Louis, Missouri, United States of America; Lund University, Sweden

## Abstract

The avian retina possesses one of the most sophisticated cone photoreceptor systems among vertebrates. Birds have five types of cones including four single cones, which support tetrachromatic color vision and a double cone, which is thought to mediate achromatic motion perception. Despite this richness, very little is known about the spatial organization of avian cones and its adaptive significance. Here we show that the five cone types of the chicken independently tile the retina as highly ordered mosaics with a characteristic spacing between cones of the same type. Measures of topological order indicate that double cones are more highly ordered than single cones, possibly reflecting their posited role in motion detection. Although cones show spacing interactions that are cell type-specific, all cone types use the same density-dependent yardstick to measure intercone distance. We propose a simple developmental model that can account for these observations. We also show that a single parameter, the global regularity index, defines the regularity of all five cone mosaics. Lastly, we demonstrate similar cone distributions in three additional avian species, suggesting that these patterning principles are universal among birds. Since regular photoreceptor spacing is critical for uniform sampling of visual space, the cone mosaics of the avian retina represent an elegant example of the emergence of adaptive global patterning secondary to simple local interactions between individual photoreceptors. Our results indicate that the evolutionary pressures that gave rise to the avian retina's various adaptations for enhanced color discrimination also acted to fine-tune its spatial sampling of color and luminance.

## Introduction

**Figure 1:**
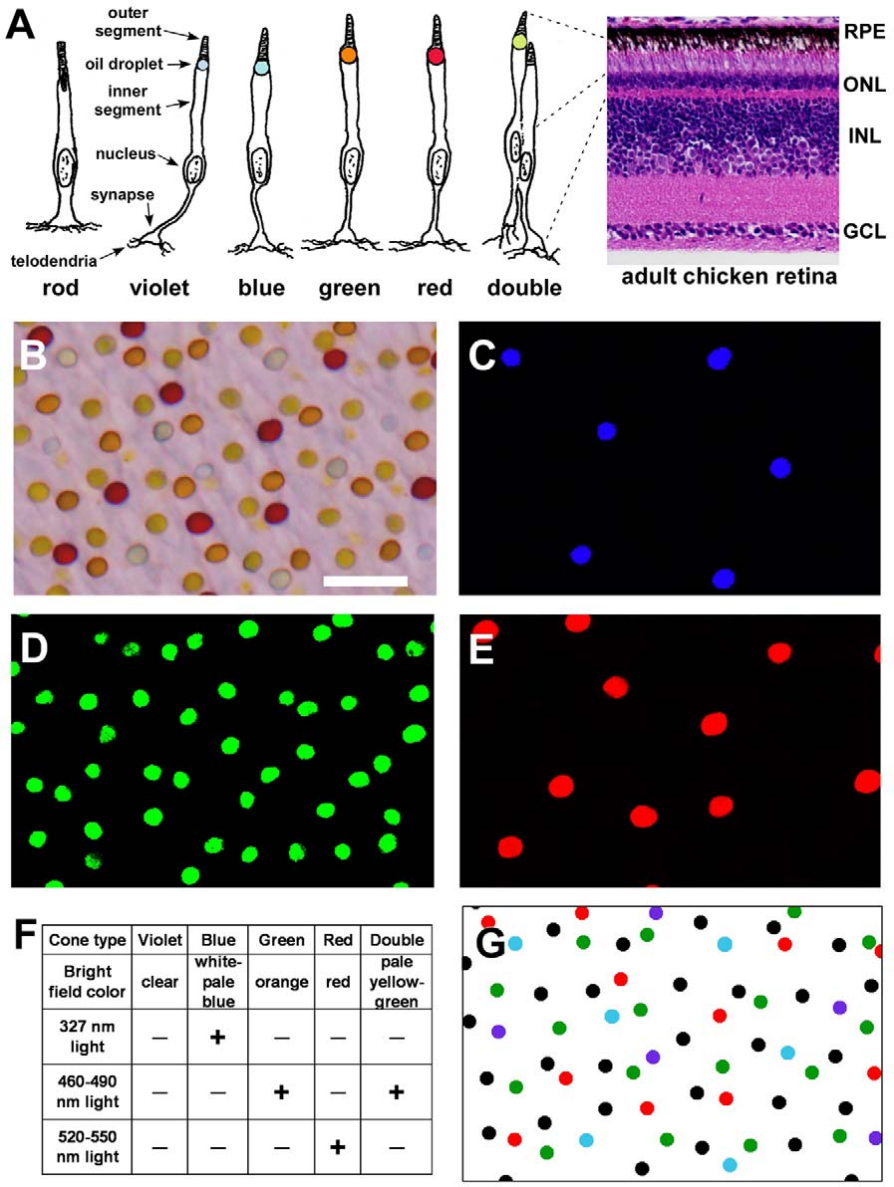
Oil droplets permit classification of chicken cone photoreceptors. (A) Diagram of the seven photoreceptor cell types of the chicken retina. Oil droplets are colored approximately according to their appearance under brightfield illumination. Rods and the accessory member of double cones lack oil droplets. A hematoxylin and eosin-stained section of an adult chicken retina is shown on the right. The drawing are based on depictions of avian rods and cones by Ramón y Cajal [73]. RPE, retinal pigment epithelium; ONL, outer nuclear layer; INL, inner nuclear layer; GCL, ganglion cell layer. (B) Brightfield view of a flatmounted P15 chicken retina viewed photoreceptor side up. Size bar = 10 µm. (C–E) Same field as in (B) viewed under ultraviolet (327 nm) light (in C), blue (460–490 nm) light (in D) and green (520–550 nm) light (in E). Only blue cones show fluorescence under ultraviolet light, and this fluorescence is short-lived. Both green cones and double cones fluoresce under blue light. Only red cones fluoresce under green light. (F) Table summarizing the appearance of chicken oil droplets under brightfield and fluorescent light. (G) Digitized versions of the field shown in (B). Colored dots correspond to their respective single cone types. Black dots represent double cones.

The chicken (*Gallus gallus*) is typical of most diurnal birds in possessing seven photoreceptor cell types including one rod and six cones (Figure 1A) [1]. Tetrachromatic color vision is mediated by four types of single cone which are maximally responsive to violet, blue, green and red light [2]. Double cones, in contrast, consist of pairs of closely apposed principal and accessory members which act as a single functional unit and are thought to mediate luminance detection that is used for motion perception [3], [4], [5]. Placental mammals lack double cones and therefore use a single set of cones for both functional purposes [6].

Prior studies have shown that most non-photoreceptor cell types in the retina tile its surface with varying degrees of regularity [7], [8], [9], [10], [11], [12], [13], [14]. This tiling reflects the need for similar, parallel processing of information across the retina [15]. Neuronal tiling is such a pervasive feature of retinas that it has been used as a defining criterion for retinal cell types [16]. Studies have shown that neurons of the same type tend to avoid each other, whereas no such avoidance is apparent between cells of different type [13]. Regular tiling is such a reliable feature of retinal cell type patterning that violation of tiling was recently used to distinguish two types of bipolar cell in the mouse, which were previously thought to represent a single cell type on account of their sharing a specific molecular marker [16]. Considerations of spatial regularity and tiling have even been used to argue that all possible bipolar cell types have now been identified in the mouse [16].

Photoreceptors display the most regular tiling of all neuronal cell types. Many teleost fish and some reptiles have almost perfectly regular ‘crystalline’ arrays of photoreceptors which occur in a variety of patterns [17], [18], [19], [20], [21], [22], [23]. The photoreceptor mosaic of zebrafish is probably the best studied example of such ‘crystalline’ arrays [21], [23], [24]. In this species, cone photoreceptors are arranged in parallel rows such that one row contains alternating pairs of red and green cones which form ‘double cones’ while the next row contains alternating blue and ultraviolet cones [21]. Adjacent rows of photoreceptors are arrayed such that blue cones are always adjacent to red cones and ultraviolet cones are always flanked by green cones [21]. This patterning between rows lends the zebrafish photoreceptor mosaic an appearance of almost crystalline regularity when viewed *en face*. Quantitative studies of the spatial regularity of the zebrafish and goldfish cone mosaics have been performed and have demonstrated a high degree of regularity which accords with the near crystalline appearance of these mosaics [21], [25].

Amongst vertebrate retinas such crystalline regularity of photoreceptors is the exception rather than the rule. The most detailed studies of mammalian photoreceptor spatial distribution to date have been performed on human and ground squirrel retinas [26], [27]. Studies in these species have demonstrated varying degrees of spatial regularity for both rods and cones. As with inner retinal cell types, photoreceptors of like type avoid the spatial vicinity of other cells of the same type but are indifferent to the presence of photoreceptors of another type. It is possible to computationally model the spatial distribution of photoreceptor mosaics by invoking simple ‘minimal spacing’ rules such that no two cells can occur within a certain defined distance of one another [26], [28]. Thus, it appears that the global regularity of photoreceptor mosaics arises due to local, homotypic interactions between individual cells. Since regular spatial sampling is critical for optimal neural reconstruction of the visual scene [29], this emergent order across the retina has clear adaptive significance.

Mounting evidence suggests that the common ancestor of modern reptiles, birds and mammals was a diurnal organism with a highly sophisticated cone visual system comparable to that of present-day birds [30], [31]. This amniote ancestor is likely to have possessed four single cones mediating tetrachromatic color vision as well as double cones for motion detection. In addition, the cones of this common ancestor are likely to have contained brightly colored oil droplets in their inner segments which are widespread amongst modern birds and reptiles [1], [30], [31], [32]. These oil droplets reside at the junction between the inner and outer segments and are thought to act as microlenses and long-pass spectral filters, focusing incoming light onto the photosensitive outer segment and improving color discrimination [32], [33], [34]. The presence of such adaptations in both modern birds and reptiles supports the hypothesis that the common amniote ancestor was a diurnal organism with a highly developed cone system [31].

During the evolution of placental mammals, many of these specialized adaptations to a strongly diurnal niche were lost [35]. The majority of placental mammals possess only two types of cones, sensitive to short- and long-wavelength light [35], [36]. In addition, placental mammals lack double cones and oil droplets [30]. The loss of multiple components of the cone visual system in these animals is thought to have occurred during a long period of nocturnality in mammalian evolution [35]. The presence of both rudimentary double cones and colorless oil droplets in marsupials and monotremes support the notion that the common mammalian ancestor did at one time possess such adaptations which were subsequently lost [30], [37], [38]. Only three clades of placental mammals have re-evolved cone-dominant retinas adapted to a diurnal niche: ground squirrels, tree shrews and primates (the lattermost showing cone dominance only within the fovea) [30]. Of these three groups, only primates have additionally evolved trichromatic color vision via duplication of the ancestral long-wavelength sensitive opsin, an event which occurred only 25 to 30 million years ago [39]. No placental mammal has successfully reacquired oil droplets [30]. Thus, despite the evolution of a variety of adaptations to the diurnal niche within these three clades, the retinas of placental mammals do not reflect the condition of the cone visual system thought to have been present in the common ancestor of amniotes.

Unlike the case of mammals, a diurnal lifestyle is presumed to have been maintained throughout the evolutionary history of birds from the common amniote ancestor [30], [31]. Thus, studies of avian cones may provide clues to the organization of the cone system of ancestral species including the most recent ancestors of birds, the theropod dinosaurs. Given the remarkable adaptations of the avian cone system for improved color discrimination, we hypothesized that the distribution of cones might be similarly optimized for spatial sampling of color and luminance. Here we show that avian cones constitute five independent but overlapping mosaics with a high degree of spatial regularity. The features of cone patterning found in the chicken are shared by a wide range of avian species suggesting that they are universal amongst birds. These results support the hypothesis that evolutionary fine-tuning of the cone system in birds extends to the level of spatial patterning.

## Results

### Avian Cone Types Can Be Distinguished by the Properties of Their Oil Droplets

In order to identify individual cone photoreceptors in the chicken retina, we took advantage of the presence of brightly colored oil droplets in their inner segments (Figure 1A,B). With the exception of those in violet cones, all cone oil droplet types contain a mixture of carotenoid pigments which endow the oil droplets with characteristic brightfield appearance and fluorescent properties (Figure 1B–F) [1], [40]. These features are identical in all cones of a given type and thus permit unequivocal classification of individual cones. Using this approach, we were able to determine the spatial coordinates of all individual cones and analyze their numbers and spatial distributions (Figure 1G and Table S1).

**Figure 2:**
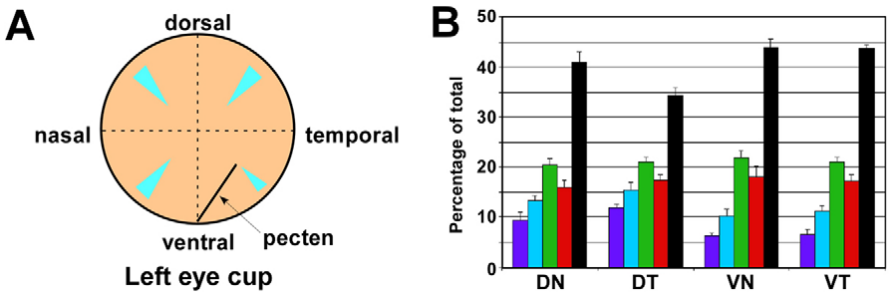
Cone photoreceptor types are present in characteristic ratios. (A) Diagram of a chicken eye cup showing the regions of the mid-peripheral retina (in light blue) from which all fields analyzed in this study were derived. (B) Percentages of cone types from each of four quadrants (n = 7 fields for each quadrant). Data for violet, blue, green and red cones are colored accordingly. Data for double cones are shown in black. Error bars indicate SD.

We examined a total of 28 post-hatch day 15 (P15) chicken retinas including seven mid-peripheral retinal fields from each of four quadrants (Figure 2A). We found that the five cone types are present in characteristic ratios as previously described (Figure 2B) [2]. In the retina as a whole double cones were the most abundant cone type (40.7%) followed by green (21.1%), red (17.1%), blue (12.6%) and violet (8.5%) single cones. Double cones were more abundant ventrally than dorsally, while blue and violet cones showed the converse pattern (Figure 2B). The density of all cone types decreased with increasing retinal eccentricity (data not shown), but the relative ratio of different cone types was nearly constant within a given quadrant.

### Individual Cone Types Tile the Retina as Highly Regular Mosaics

**Figure 3:**
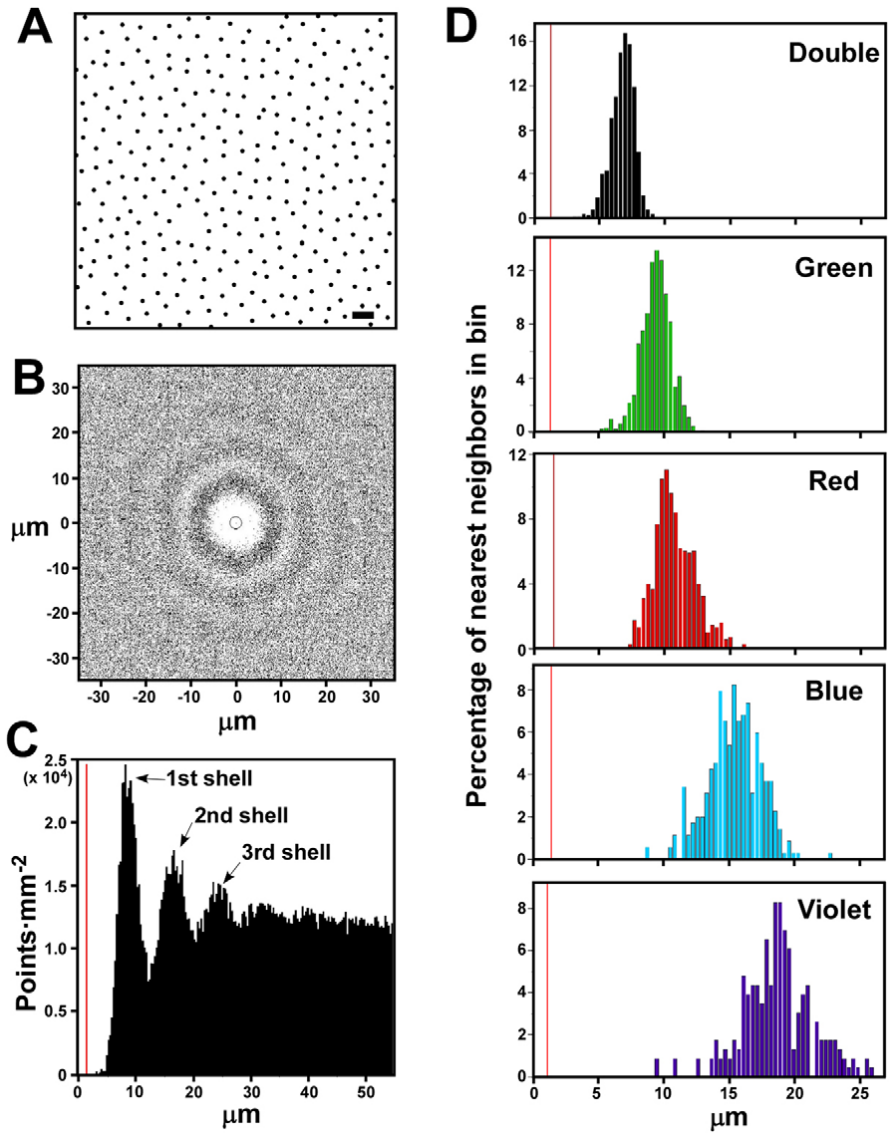
Cone photoreceptors tile the retina as five overlapping mosaics. (A) Digitized image of double cone distribution in a portion of a single field (dorsal-nasal field 7 in Table S1). Size bar = 10 µm. (B) Spatial autocorrelogram for entire field of double cones of which a portion is shown in (A). The circle around the origin indicates the diameter of an average double cone oil droplet. (C) Density recovery profile derived from the spatial autocorrelogram in (B). The peaks designated “1^st^ shell” etc. are explained in the main text. The vertical orange line indicates the average diameter of a double cone oil droplet. (D) Distribution of nearest neighbor distances for each of the five cone types within a single retinal field (dorsal-nasal field 7 in Table S1). The vertical orange line indicates the average diameter of the oil droplet corresponding to each of the indicated cone types.

When a field of retinal oil droplets is viewed as a whole, there is little apparent order. However, when cone types are considered individually, they show a highly regular distribution with a relatively uniform distance between neighboring cones (Figure 3A). In order to evaluate this regularity systematically, we created spatial autocorrelograms for each of the cone types (Figure 3B and Figure S1) [26]. In this analysis, each cone in a field is placed at the origin of a coordinate system and all other cones are replotted relative to that point. This process is then repeated for all cones in the field. The resultant graph for double cones shows a circular region immediately surrounding the origin which is virtually devoid of points (Figure 3B). This finding indicates the presence of an ‘exclusion zone’ around individual cones of a given type within which cones of the same type are only rarely encountered. Similar exclusions zones are present around each of the single cone types as well (Figure S1).

Progressing farther out from the origin of the autocorrelogram there occur alternating shells of increasing and decreasing cone density which can be better appreciated by graphing the data as a density recovery profile (DRP; Figure 3C) [41]. The DRP depicts the spatial density of cones at progressively greater distances from the origin of the autocorrelogram. It shows a region of very low cone density in the immediate vicinity of the origin followed by a series of density peaks at progressively greater distances from it (Figure 3C). Each successive ‘shell’ beyond the first represents the nearest neighbors of individual cones progressively more removed from the primary cone at the origin (Figure 3C). The presence of these successive shells is indicative of long-range order within the double cone mosaic that extends beyond the nearest neighbors of a given photoreceptor. Such long-range order is also evident in the single cone mosaics but to a lesser extent (Figure S1).

The distances of all the nearest neighbors of a given cone type follow an approximately Gaussian distribution (Figure 3D). Within a given field, the mean nearest neighbor distance is different for each cone type, and there is a strong correlation between the mean nearest neighbor distance and the width of the distribution around the mean (r = 0.94) (Figure 3D). These data indicate that homotypic cone spacing does not involve an absolute ‘exclusion radius’ within which cones of the same type never occur. Rather, there is a preferred distance at which cones of the same type position themselves relative to one another.

### Cone Mosaics Display a High Degree of Topological Order

**Figure 4:**
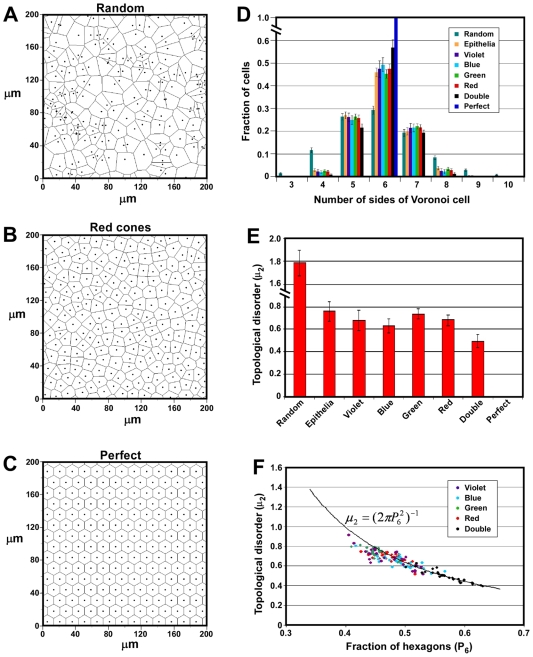
Cone mosaics show a high degree of topological order. (A–C) Voronoi tessellations of a portion of a red cone field (B) and a random (A) and perfect (C) distribution of points of the same density as in (B). (D) Graph showing the average P_n_ distributions for all chicken cone types as well as simulated random and perfect distributions. ‘Epithelia’ indicates the average P_n_ distribution for five different animal and plant epithelia as given in [Ref. 38]. Note that the P_n_ distribution for the random simulations included a small number of 11-, 12- and 13-sided cells which are not shown. Error bars are SD. (E) Graph showing the topological disorder (μ_2_) for all five cone types as well as random and perfect distributions. ‘Epithelia’ are as described in (D). Error bars are SD. (F) Graph of P_6_ vs. topological disorder (μ_2_) for all 140 P15 cone mosaics examined. The solid curve indicates the value of Lemaître's law (equation shown in the graph) in the range, 0.34<P_6_<0.66.

One way of assessing topological order in a two-dimensional (2D) distribution of points is to use Voronoi tessellations [42]. In this kind of tiling, all points in the plane are partitioned into Voronoi domains which represent all those points in the plane that are closer to a particular cell than to any other cell. We have created Voronoi tessellations for all of our cone photoreceptor distributions in order to derive quantitative measures of their topological order (Figure 4A–C and data not shown). It can be seen in a typical field that red cones tile the plane in a highly regular fashion which has a degree of orderliness between that of a random distribution of equal density and a perfect hexagonal array of the same density (Figure 4A–C). For comparison with the cone distributions, we created Voronoi tessellations for one hundred random distributions of points and calculated the fraction of polygons of each type that were observed (Figure 4D). The highest fraction of polygons were hexagons (P_6_ = 0.293±0.018 [mean ± SD]), but there was a wide distribution of sizes ranging from 3-sided up to 13-sided. As the degree of order in a Voronoi tiling increases, there is a corresponding increase in P_6_ and a decrease in the width of the polygon distribution. In the limiting case of a perfectly regular tiling, P_6_ = 1 (Figure 4D).

We found that Voronoi tilings of the four single cones all showed very similar polygon distributions with P_6_ ranging from 0.454±0.019 (mean ± SD) for green cones up to 0.494±0.031 (mean ± SD) for blue cones (Figure 3D). Strikingly, a wide range of post-mitotic animal and plant epithelia show polygon distributions very similar to those observed here for chicken single cones (Figure 4D) [43], [44]. The mean P_6_ value for epithelia from five different species was 0.460±0.020 (mean ± SD) [44]. It has been suggested that epithelia from diverse species converge on this particular polygon distribution as a topological consequence of the cell division process [43]. It is therefore intriguing that the Voronoi tilings of all four single cone types match this distribution so closely, since these tilings are only notional epithelia and the constituent cells used to generate them are not spatially contiguous. It is possible that the degree of order observed in the individual single cone mosaics reflects the orderliness of the underlying epithelium of which they are a part. However, double cones were found to have a polygon distribution quite different from single cones with P_6_ = 0.570±0.034 (mean ± SD) (Figure 4D). This finding demonstrates a higher degree of topological order in double cones than single cones and suggests that the orderliness of a given cone type is not a necessary consequence of the degree of order in the underlying epithelium.

An alternative measure of topological regularity is the variance of the probability distribution *P_n_* of the number *n* of sides of a given cell [45], [46], [47]:
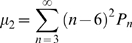
Whereas P_6_ serves as a measure of order, μ_2_ is a measure of the spread of the polygon distribution and is therefore a measure of topological disorder. We found that the four single cone types have similar μ_2_ values ranging from 0.634±0.060 (mean ± SD) for blue cones up to 0.734±0.046 (mean ± SD) for green cones (Figure 4E). The single cone values are again comparable to that found for multiple epithelia 0.760±0.086 (mean ± SD). In contrast, double cones showed a degree of disorder significantly less than single cones 0.494±0.063 (mean ± SD). Thus, two different measures of topological orderliness demonstrate a high degree of order in all cone types, with double cones showing a higher degree of order than single cones. The two functional classes of cones in the chicken retina, single cones which subserve color vision and double cones which mediate motion perception, therefore form two distinct classes with respect to topological order.

Next, we studied the relationship between P_6_ and μ_2_ in the chicken cone mosaics. It has been shown that a wide range of 2D cellular mosaics found in nature including examples from metallurgy, geology and ecology as well as mosaics obtained from experimental and computational simulations all obey a quasi-universal topological relation between P_6_ and μ_2_ known as Lemaître's law [48], [49], [50]. In the range, 0.34<P_6_<0.66, this law takes the form:
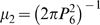
In order to assess whether the chicken's cone mosaics also obey this law, we plotted P_6_ versus μ_2_ (Figure 4F). Those mosaics that had P_6_>∼0.47 showed μ_2_ values which were in close agreement with Lemaître's law (Figure 4F and Figure S2). In contrast, mosaics with a P_6_<∼0.47 tended to have a value for μ_2_ which was less than would be predicted by the law. The near universality of this law is thought to be a consequence of the fact that all mosaics which obey it are statistical ensembles in equilibrium [50]. The deviation of some cone mosaics from this law at lower P_6_ values therefore suggests that these mosaics may not be in statistical equilibrium. Alternatively, there may be unknown biological constraints which contribute to this deviation.

### Individual Cone Mosaics Are Spatially Independent of One Another

**Figure 5:**
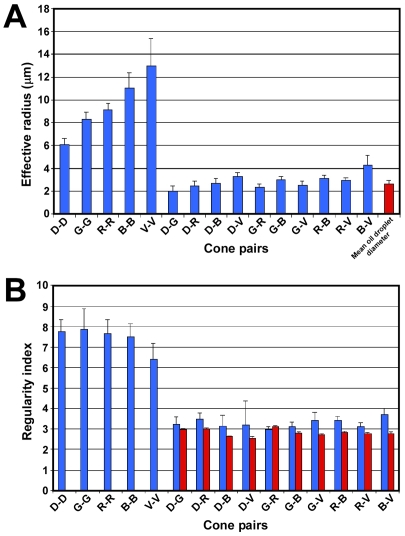
Cone mosaics are spatially independent. (A) Graph of the effective radius between cones of the same type (homotypic pairs) and different types (heterotypic pairs). Also shown for comparison is the average oil droplet diameter for all cone types. ‘D-D’, ‘Double cone-Double cone’; ‘G-G’, ‘Green cone-Green cone’ etc. Error bars are SD. (B) Graph of the nearest neighbor regularity indices for cones of the same type (homotypic pairs) and different types (heterotypic pairs) (blue bars). Also shown are regularity indices for simulated mosaics as described in the main text (red bars). Abbreviations are as in (A). Error bars are SD.

**Figure 6:**
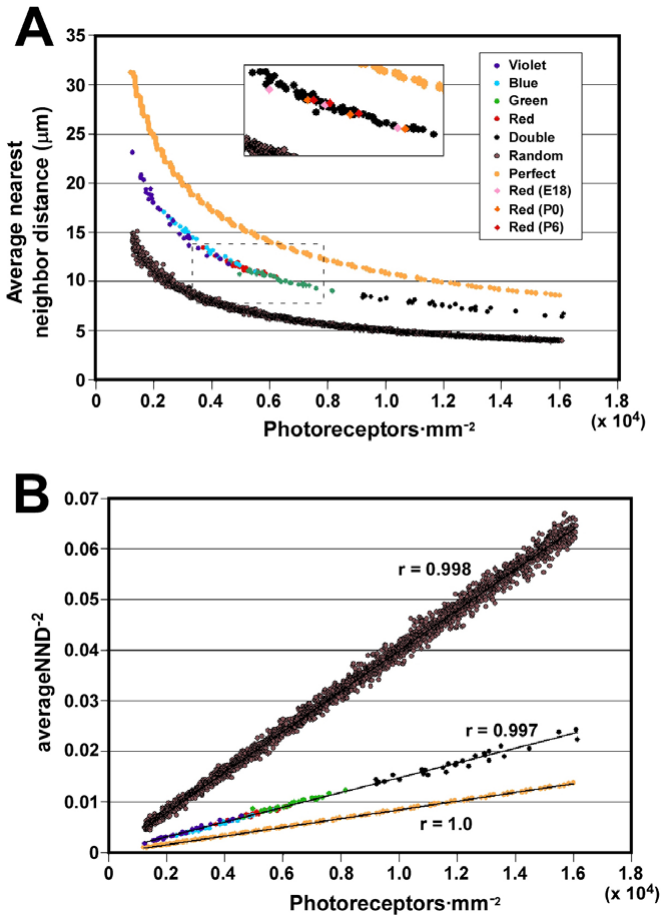
All cone types measure intercone distance with the same yardstick. (A) Graph of photoreceptor density vs. average nearest neighbor distance for all 140 P15 cone mosaics examined (middle curve). The upper and lower curves are graphs of density vs. average nearest neighbor distance for a series of computer-generated perfect and random distributions, respectively. The inset shows data for the three developmental timepoints (i.e., red cones at E18, P0 and P6). It corresponds to the region of the main graph highlighted with a dotted box except that all P15 chicken datapoints shown in the main graph are shown in black to facilitate visualization of the developmental timepoints. (B) Graph of the same datapoints as in (A) but shown as density vs. the inverse-square of the average nearest neighbor distance. The linear correlation coefficients (r) for the best fit line for each of the three datasets are shown.

Given the homotypic spacing observed between cones of the same type, we wished to determine whether similar spacing occurs between cones of different type. We evaluated whether there was any tendency for heterotypic pairs of photoreceptors to repel one another by measuring the effective radius of exclusion (ERE) around individual photoreceptors (Figure 5A). The ERE is a measure of the zone around individual photoreceptors within which there is a deficiency of other photoreceptors [11], [41]. If heterotypic pairs of photoreceptors have no tendency to repel one another one would expect that their proximity should only be limited by the size of the individual cells. In such a case, one would expect that the ERE should be roughly equal to one cell diameter, since when two cells directly abut one another their centers are one cell diameter apart. The ERE for all homotypic pairs of cones was significantly greater than one cell diameter and correlated closely with that cell type's average nearest neighbor distance (Figure 5A; compare Figure 6A). In contrast, the ERE for all heterotypic pairs was comparable in size to the average diameter of an oil droplet which we used as a surrogate measure of photoreceptor diameter (Figure 5A; also see Figure 1A). These data suggest that heterotypic pairs of cones do not repel one another, and that their proximity is only limited by the size of their cell bodies.

In order to further explore the possibility of spatial co-regularities between cone mosaics we employed a commonly used measure of geometric order within cellular mosaics known as the regularity index (RI). The RI is equal to the average nearest neighbor distance divided by its standard deviation [10], [26]. Before evaluating the RI for heterotypic pairs of cones, we determined the RI for the five homotypic cone mosaics. We found that red, green and blue single cones and double cones all had very similar RIs ranging from ∼7.5 to 7.8 (Figure 5B). Violet cones had a slightly lower mean regularity index of ∼6.4. For comparison, a prior study of the rod and S-cone mosaics of the ground squirrel retina found mean RIs of 2.8 and 4.5, respectively [26]. In addition, studies of retinal ganglion cell mosaics have found RIs ranging from ∼3.0 to ∼6.5 [7], [9], [11]. The chicken cone photoreceptor mosaics therefore appear to be more regular than most other previously characterized mosaics of retinal neurons.

Next, we determined the RI for all possible heterotypic (X-Y) pairs of cone by identifying the nearest ‘Y’ neighbor of every ‘X’ cone and then calculating the mean and standard deviation (Figure 5B). We found that the RIs for all heterotypic pairs were significantly less than for homotypic pairs and fell between ∼2.9 and 3.7. Although lower than the values found for homotypic pairs, these RIs are still larger than what would be expected for a totally random distribution of points (∼1.9). This discrepancy can be accounted for by two possible factors. First, since real photoreceptors occupy space, their possible locations are constrained by the fact that two photoreceptor cannot lie on top of each other. This constraint will therefore limit the possible distribution of individual photoreceptors and thereby increase their regularity. Secondly, since both the ‘X’ and ‘Y’ photoreceptor mosaics are highly ordered, apparent co-regularities might occur simply due to the fact that the two ordered mosaics happen to fall in register with each other.

In order to control for the effects of steric hindrance and spurious co-regularity due to random spatial registration of mosaics, we carried out computer simulations to assess their effects. We generated random distributions of photoreceptor ‘Y’ that matched the density and mean regularity index of the real ‘Y’ mosaics using a sequential addition, ‘hard disk’ model (see Materials & Methods for details) [26], [51], [52]. We then calculated the RI for the heterotypic pairs X-Y by using the coordinates of the real ‘X’ cells and comparing them to the simulated ‘Y’ mosaics. This simulation was carried out one hundred times for all possible heterotypic pairs, and the mean and standard deviation of the resultant RIs were determined (Figure 5B). These simulations produced RIs which were comparable to what was found for the real X-Y pairs but with somewhat lower mean values (red bars in Figure 5B). This result suggests that the RIs we found for the real heterotypic pairs reflect the degree of co-regularity to be expected between two independent, but highly ordered mosaics [14]. We therefore conclude that if any higher-order spatial correlations exist between cone types, they are likely to be quite subtle. The spatial independence of the individual cone types accords well with what has been previously reported for a range of different retinal cell types [13], [14], [16].

### All Cone Types Use the Same Yardstick for Measuring Intercone Distance

In order to obtain further insights into the mechanism of cone spacing, we plotted the average nearest neighbor distance between cones of the same type as a function of photoreceptor density. We found that average nearest neighbor distance decreases as a function of increasing density (Figure 6A). Figure 6A shows 140 distinct datapoints (five photoreceptor types x 28 fields) which vary over a greater than ten-fold range of densities. Individual photoreceptor types show a range of densities on account of the fact that sample fields derive from various retinal eccentricities and quadrants. For comparison with the real data, we also plotted data from computer-generated random and perfect distributions of varying density (Figure 6A). Both the perfect and the random distributions follow curves which are very similar in shape to that of the real data but which are shifted up and down, respectively, along the Y-axis. Thus the curve describing the chicken's cone photoreceptors mosaics resides at an intermediate position between those for random and perfect distributions. The position of this curve can therefore be used to quantify the degree of geometric order in the mosaics. We will return to this point below. First, it should be noted that data from all five cone photoreceptor types appear to fall on the same curve (Figure 6A). Thus, at any given density, the average nearest neighbor distance is independent of photoreceptor type. This observation contrasts with the earlier conclusion that all five photoreceptor types are spatially independent and are therefore likely to have distinct molecular mechanisms of homotypic spacing. Instead, it suggests that they share a common mechanism, at least with respect to determining the magnitude of the spacing between cells.

In order to assess at what point in development the orderliness of the cone mosaics first appears, we determined the spatial coordinates of red cones at three earlier developmental timepoints: embryonic day 18 (E18) and post-hatch days 0 and 6 (Table S1). We then plotted density versus average nearest neighbor distance. We found that at all timepoints examined, the data fall on the same curve as the data from P15 (inset in Figure 6A). Since oil droplet pigmentation first becomes apparent in the peripheral retina around E16–17 and full pigmentation of all the red cones first appears somewhat later [53], E18 was the earliest point at which we could reliably distinguish all cones of this type. We therefore conclude that, at least for red cones, the adult pattern of spatial organization is already achieved at the earliest point at which oil droplets can be distinguished.

### A Single Parameter Defines the Regularity of All Five Cone Mosaics

In order to quantify the degree of geometric order inherent in the cone photoreceptor mosaics, we next plotted density versus the inverse-square of the average nearest neighbor distance. It can be seen that all of the real datapoints as well as the simulated random and perfect distributions fall on three straight lines with different slopes (Figure 6B). If extended to the Y-axis, all three lines can be seen to pass through the origin since as density → 0, average nearest neighbor distance → ∞ (Figure 6B). Because all three lines are of the form, y  =  mx, the slope, m, can be used as a measure of the degree of order within the cone mosaics as a whole. Thus, remarkably, it is possible to reduce the spatial organization of all five cone photoreceptor mosaics to a single quantitative measure of geometric order.

### Similar Mechanisms of Cone Spacing Are Used by a Wide Range of Bird Species

**Figure 7:**
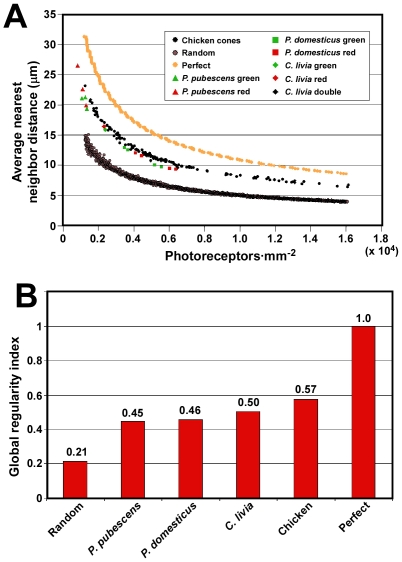
A range of bird species show similar cone patterning. (A) Graph of photoreceptor density vs. average nearest neighbor distance for three additional species of bird representing three different orders. All P15 chicken datapoints are shown in black for clarity. *P. pubescens*, *Picoides pubescens*; *P. domesticus*, *Passer domesticus*; *C. livia*, *Columba livia*. (B) Graph of the global regularity indices for all four bird species examined as well as for computer-generated random and perfect distributions. The global regularity index is the inverse of the slope of the best fit linear curves of the form, y  =  mx, for each of the datasets as shown in Figure S3. All values are normalized to that for perfect which is set equal to one.

Next, we wished to determine the generality of the relationship between density and average nearest neighbor distance amongst birds. We therefore examined the spatial distribution of a subset of cones from three additional species belonging to three different orders: downy woodpecker (*Picoides pubescens*), house sparrow (*Passer domesticus*) and pigeon (*Columba livia*) (Table S1). We found that these three bird species show a relationship between density and average nearest neighbor distance very similar to that of the chicken (Figure 7A and Figure S3). In order to assess the degree of order within the cone mosaics of these species we replotted the data from each as density versus the inverse-square of the average nearest neighbor distance (Figure S3). We then fit each dataset with a straight line of the form, y  =  mx, in order to estimate the degree of geometric order within their cone mosaics and compare the values to that of chicken. To simplify comparison between species we derived a global regularity index which is the inverse of the slope, m, normalized to the value of a perfect hexagonal array set equal to one. The photoreceptor mosaics of *P. pubescens*, *P. domesticus* and *C. livia* have global regularity indices of 0.45, 0.46 and 0.50, respectively, compared to the chicken which has 0.57 (Figure 7B). The chicken mosaic therefore appears to be somewhat more orderly overall than those of the other species. Yet, given the relatively small number of datapoints for the other species, these values must be considered tentative. Nevertheless, since the cone mosaics from four species representing four orders of bird show such similar geometric features and spatial organization, it is likely that they share similar mechanisms of cone spacing which may be representative of all diurnal bird retinas.

## Discussion

In this study we have used the colored oil droplets present in the inner segment of cone photoreceptors to characterize the spatial distribution of the chicken's five functional classes of cone. We found that each type of cone is arrayed as a highly regular mosaic with a characteristic spacing between cones of the same type. All five cone mosaics display a high degree of topological and geometric order but are spatially independent of one another. Remarkably, all cone types use a similar density-dependent yardstick to measure intercone spacing. Based on the relationship between density and nearest neighbor distance, we derived a single parameter that uniquely characterizes the regularity of all the cone mosaics within the retina. The value of this parameter was determined for three additional species of bird, which were found to have cone spatial patterning which was fundamentally similar to that of the chicken. This result suggests that the principles of cone spacing identified in the chicken may be universal among diurnal birds. These results confirm that avian cone photoreceptor have an extremely high degree of spatial organization which is likely the result of evolutionary selection.

In evaluating the spatial distribution of chicken cones, we found that as photoreceptor density decreases, the average nearest neighbor distance between cones of the same type increases. The net result of this scaling is that photoreceptors maintain a relative uniform degree of spatial regularity despite changes in density. A similar scaling relationship between density and average nearest neighbor distance was previously found for a type of ganglion cell in the chicken [11]. In contrast, another study in the ground squirrel found that rods and S-cones maintain a ‘minimal distance’ between cells of the same type which is constant throughout the retina, independent of cell density and specific to each cell type [26]. Thus, at a given density, the S-cone mosaic in the ground squirrel is more regular than the rod mosaic since it maintains a greater ‘minimal distance’ between neighbors of the same type. One consequence of this lack of scaling is that the regularity of the ground squirrel's rod mosaic decreases with decreasing rod density [26]. These findings suggest that the scaling relationship between photoreceptor density and the average nearest neighbor distance we observed in the chicken retina is not a feature of all photoreceptor mosaics. Furthermore, it is clear that even within a single retina, not all photoreceptor types necessarily measure intercone distance in the same manner. One possible explanation of this result is that rods and cones may differ in their mechanisms of spacing. Since chicken rods lack oil droplets, their spatial distribution was not analyzed in the present work, and so this question could not be addressed. Yet, prior studies in chicken suggest that rod density is roughly equal to that of double cones in the peripheral retina and that they are highly regular in their distribution [54], [55]. Future studies will address whether chicken rods obey the same rules as cones with respect to homotypic spacing.

The degree of order within two-dimensional cellular mosaics can be characterized by the distribution of values for the area of individual cells within the mosaic (geometric order) or by the distribution of values for *n*, the number of neighbors each cell has (topological order) [45], [56]. Measures of topological and geometric order are often found to be strongly correlated in a wide variety of organic and inorganic 2D cellular mosaics [56]. Nevertheless, the two measures of order are mathematically independent and do not necessarily correlate [56], [57], [58]. We found that all four single cone types in the chicken have a similar degree of topological order as defined by their P_n_ functions (see Figure 4D). In contrast, double cones showed a distinctively higher degree of topological order than single cones. One measure of geometric order, the nearest neighbor regularity index, showed similar values for double and single cones (Figure 5B and data not shown). Thus, in the case of double cones, there appears to be a dissociation between the two types of order. The reason for the seemingly disproportionate degree of topological order found in the double cone mosaics of the chicken is currently unknown. However, given the putative role of double cones in luminance detection and motion perception [6], this high degree of order could represent an evolutionary adaptation for these functions.

A wide range of post-mitotic animal and plant epithelia show very similar P_n_ functions and hence display similar degrees of topological order [43], [44]. The presence of a similar P_n_ function in such a wide range of epithelia has been posited to arise as a topological consequence of mitosis [43]. Surprisingly, we found that the P_n_ functions of the Voronoi tessellations of single cone mosaics in the chicken are similar to those found in epithelia (Figure 4D). This finding is difficult to explain in terms of mitosis since these Voronoi tessellations do not represent actual epithelia. However, since all of the individual cone mosaics reside in the same epithelium, it is possible that their P_n_ functions simply mirror that of the underlying epithelium. If the underlying epithelium had a P_n_ function similar to the one observed in many other epithelia, then a random assignment of cells (i.e., polygons) from the underlying epithelia to each of the individual cone mosaics would, on average, endow the individual mosaics with a similar P_n_ function. However, we know that the individual cone mosaics do not represent random samplings of the underlying epithelium. Furthermore, the fact that double cones have a very different P_n_ function argues against this simple interpretation. These findings suggest that there may be unknown biological reasons for the repeated occurrence of this particular P_n_ function.

The results of the present study constrain the range of possible models that can explain the formation of the chicken's cone mosaics. Any model of mosaic formation must encompass two key aspects of cone photoreceptor patterning. On the one hand, the five cone mosaics are spatially independent and show no evidence of heterotypic repulsion between different cone types. These facts suggest the existence of distinct biochemical mechanisms of spacing unique to each cone type. On the other hand, cone-to-cone spacing, although density-dependent, is independent of cell type, suggesting a mechanism of measuring intercone distance which is shared by all cone types. If cone spacing is established simultaneously or in temporally overlapping waves for the five cone types, it seems necessary to invoke multiple distinct molecular signals mediating homotypic interactions for each of the five types. Such interaction could be mediated either by a diffusible signal or by cell-cell contact. In this scenario, cone-spacing might involve a ‘two-component’ mechanism consisting of a cone type-specific signaling system mediating cell type recognition and a second shared system for measuring the distance between cones.

**Figure 8:**
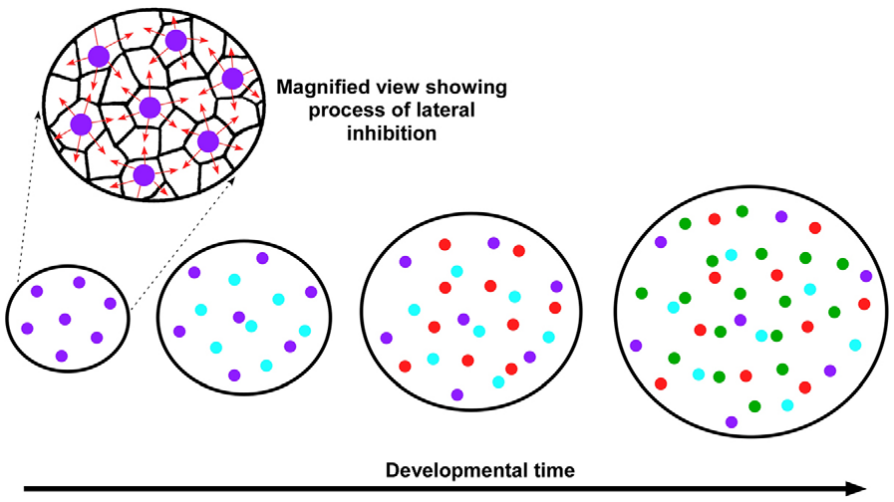
A model for the formation of the photoreceptor mosaics of the chicken. In this model the individual photoreceptor types establish their spacing in a series of temporally discrete waves. The least abundant photoreceptor type (i.e., violet cones) establishes spacing first, possibly via a lateral inhibition mechanism (far left). Then, the next most abundant photoreceptor type, blue cones, establishes its spacing. This process continues until spacing has been established for all photoreceptor types (the diagram only shows the four single cone types). The addition of subsequent waves of photoreceptors results in a relatively uniform expansion of the epithelium and a concomitant ‘spacing out’ of those photoreceptor types whose spacing was established earlier. Since spacing is established in discrete steps, all photoreceptor types can, in principle, employ the same biochemical mechanism to establish spacing.

If spacing occurs in temporally separate waves for the different types of photoreceptors, it is possible to posit models which involve only a single biochemical mechanism for all photoreceptor types (Figure 8). In such a model, the least abundant photoreceptor type (i.e., violet cones) would establish spacing first, perhaps via a lateral inhibition mechanism such as Notch-Delta signaling [59], [60]. Once violet cone spacing is complete, these cells would turn off expression of the molecules mediating lateral inhibition, and the next most abundant cell type (blue cones) would establish spacing using the same mechanism. This process would be repeated until all cells types had established their spacing. Uniform growth of the photoreceptor epithelium would result from addition of successive generations of cells via cell division or intercalation (Figure 8). This model, although purely theoretical, has the advantage of requiring only a single biochemical mechanism to establish homotypic spacing. In addition, the progressive expansion of the photoreceptor epithelium would result in the observed relationship between average nearest neighbor distance and cell density as depicted in Figure 6A. Furthermore, local non-uniformities in the expansion of the epithelium over developmental time would be predicted to result in progressively greater variation in nearest neighbor distances for those cell types whose spacing was established earlier, a fact which we observed in the chicken retina (see Figure 3D).

A variety of models and theoretical mechanisms have been proposed to explain the development of the nearly crystalline cone photoreceptor mosaics of certain teleost fish species [21], [24], [61], [62], [63]. In one computer modeling study [63], the author used a ‘cell rearrangement’ algorithm to reproduce the ‘row mosaic’ arrangement of cone photoreceptors found in the zebrafish retina. In this model, cells of different types were at first randomly arrayed on a lattice and then allowed to exchange positions with adjacent cells so as to optimize inter-cell adhesion. The author found that it was possible to arrive at an arrangement similar to that of the zebrafish mosaic given the correct choice of homo- and heterotypic ‘adhesion’ strengths. Not surprisingly, given the regular spatial relationship between heterotypic pairs of cones in the zebrafish, it was found that heterotypic adhesion played a more important role than homotypic adhesion in establishing the mosaic. Given that the mosaics of different cone types appear to be independent in the chicken, it is unlikely that such heterotypic adhesive interactions are active during avian retinal development.

Another model for the formation of the zebrafish cone mosaic posits that cell-cell signaling between differentiating photoreceptors and adjacent undifferentiated progenitors may be responsible for patterning [21]. In this model, a cohort of presumptive cones are born in a linear pattern and then individual cone sub-types differentiate sequentially along a moving front with red cones differentiating first followed by green, blue and ultraviolet [21]. In this scenario, the first-born red cones instruct the adjacent undifferentiated progenitors via cell-cell signaling to assume defined fates. This process is then repeated until all of the sub-types are generated in the correct spatial distribution. Apropos of this model, it has been shown that disruption of Notch-Delta signaling in the developing zebrafish retina results in marked defects in the planar patterning of photoreceptors such that the regular mosaic pattern is lost and photoreceptors of the same type show a highly irregular and partially clumped distribution [64]. This finding suggests that Notch-Delta signaling may represent one of the mechanisms whereby this patterning is established in zebrafish.

The remarkable regularity of the chicken's cone mosaics raises the question of its adaptive significance. Theoretical analyses have suggested that optimal spatial sampling of the visual scene is achieved by perfectly regular, hexagonal arrays of receptors and that any deviation from this pattern results in a decrement in the quality of the reconstructed image [29], [65]. If we assume that regular spatial sampling is critical for the survival of highly visual species such as birds and primates, one may ask why they have not evolved perfectly ordered ‘crystalline’ photoreceptor mosaics. One interesting explanation that has been offered is that a modest degree of irregularity within the photoreceptor mosaic can actually serve to reduce the amount of spatial aliasing that occurs when visual scenes are sampled by perfect arrays of photoreceptors [66], [67]. However, others have argued that the strongly periodic patterns which are prone to aliasing are not frequent in the normal visual environments of most vertebrates and would therefore probably be insufficient to account for the evolution of disordered cone patterning [68].

Another potential explanation for cone disorder is that it may be topologically impossible to pack six perfectly hexagonal photoreceptor mosaics (i.e., five cone and one rod mosaic) within a single epithelium. The question then arises whether the photoreceptor mosaics of the chicken retina are as regular as they can be given the ratios of their occurrence and these packing constraints. Under such conditions, any increase in the regularity of one mosaic might necessitate a decrement in the regularity of one or more of the other mosaics. Thus, although the individual cone mosaics are spatially independent, their regularities may depend on packing constraints within the photoreceptor epithelium and therefore be interdependent. Given the ratios and densities of its photoreceptors, it is possible that the chicken's mildly disordered photoreceptor mosaics represent an optimal solution to a 2D packing problem [69]. Future computational modeling studies will be required to address this question.

It has been postulated based on a variety of theoretical considerations that birds use two separate sets of photoreceptors for detection of chromatic and luminance signals, the single cones and double cones, respectively [5], [6]. Primates, in contrast, jointly sample color and luminance information through the same set of photoreceptors [6], [70], [71]. Specifically, primate luminance detection mechanisms combine the outputs of both red and green cones whereas all three cone types mediate detection of chromatic signals [6], [71]. Given the apparent importance of regular photoreceptor spacing in the visual ecology of birds, it is surprising that the red and green cone mosaics of the human fovea are nearly random in their distribution [71]. In fact, in those foveae where departures from randomness were noted, there was evidence of modest clumping of cones of the same type [71]. It has been hypothesized that the nearly random arrangement of red and green cones in humans could represent a compromise between the demands of color and luminance detection [71]. In an organism that jointly samples color and luminance, some degree of photoreceptor clumping may actually benefit color vision in the peripheral retina as well as high acuity spatial vision [71]. Yet such benefits come at the cost of confusing spatial and spectral information at small spatial scales [71].

The channeling of spectral and spatial signals through the same set of photoreceptors may also help explain the absence of colored oil droplets in primate retinas. Although oil droplets improve color discrimination, they reduce photoreceptor sensitivity [34]. It is therefore possible that the detrimental effects of decreased sensitivity on spatial vision could outweigh the benefits of improved color discrimination that oil droplets would confer in these species. Thus, the absence of regular spatial patterning of red and green cones and the failure to re-evolve oil droplets in primates might be a simple consequence of the detection of spectral and spatial information by the same photoreceptors. Correspondingly, separation of these two information channels in the ancestors of birds may have been the evolutionary innovation which permitted the subsequent elaboration of additional cone adaptations such as the regular spatial patterning documented in the present study.

## Methods

### Analysis of Cone Photoreceptor Distribution

All animals studies were conducted in accordance with the Guide for the Care and Use of Laboratory Animals and the Animal Welfare Act and were approved by the Washington University in St. Louis Institutional Animal Care and Use Committee. Post-hatch chickens (*Gallus gallus*, White leghorn; Charles River Laboratories; North Franklin, CT) were euthanized via carbon dioxide asphyxiation, and embryonic chickens were euthanized by decapitation. Eyes were removed from the head by blunt dissection, and the anterior segment was cut off with a razor blade. The vitreous body was removed and the eyecup was incubated for 30 minutes at 37°C in Hank's Buffered Saline Solution with calcium and magnesium to facilitate separation of the retina from the retinal pigment epithelium (RPE). The retina was oriented as shown in Figure 2A, with the inferiormost extent of the pecten oculi defining ventral. RPE-free portions of mid-peripheral retina were removed with iridectomy scissors and fixed for 30 minutes in freshly prepared 4% paraformaldehyde in 1X phosphate-buffered saline solution (PBS). The chicken retina is prone to stretching which can result in an abnormal, elliptical exclusion zone in the spatial autocorrelogram. For this reason, great care was taken at all stages of handling the retinal tissue to minimize trauma. In addition, all fields found to have elliptical exclusion zones were excluded from further analysis. After fixation, the retinal fragment was rinsed several times in PBS and flatmounted, photoreceptor side up, on a glass microscope slide in a drop of PBS. Fragments of glass coverslip (0.16–0.19 mm thick) were placed around the specimen as ‘legs’ to support an intact coverslip which was then laid on top. Images of the retina in three different planes of focus at the level of the oil droplets were taken at 400× magnification under brightfield illumination on a compound microscope (Olympus BX51) equipped with a CCD camera (Olympus DP70). Additional images were then captured under illumination with ultraviolet (327 nm), blue (460–490 nm), and green (520–550 nm) light. The fluorescent images were post-processed in Photoshop to maximize contrast. Individual photoreceptor types were identified by overlaying the various brightfield and fluorescent images in Photoshop. The position of the individual cones was then recorded manually by placing colored dots of uniform size on a different layer for each cone type. ImageJ software (NIH) was used to define the center of mass for each dot in a field and the X,Y coordinates of all dots were recorded.

A single individual of each of three additional species (*Picoides pubescens*, *Passer domesticus*, and *Columba livia*) was found in a moribund state. Immediately upon the death of the animal, retinas were processed as for the chicken. Fields from both eyes and in some cases at varying eccentricities were analyzed. Since the fluorescent properties of these species' retinas were somewhat different those of the chick, it was only possible to distinguish a subset of cone types with certainty using brightfield illumination.

### Voronoi Tessellations

All computational analyses and calculations were performed using custom Matlab scripts and Microsoft Excel. Voronoi tessellations of photoreceptor distributions were created with a custom script using a Matlab function called ‘Voronoi’. In order to avoid edge effects, only those Voronoi cells whose vertices all lie within the field were included in subsequent analyses. P_n_ distributions were calculated from the number of vertices of the individual Voronoi cells of a given photoreceptor distribution.

### Spatial Distribution Analyses

Nearest neighbor analysis, spatial autocorrelograms, density recovery profiles and effective radii were all calculated as described previously [10], [11], [26], [41], [72]. In the nearest neighbor analysis, the distance from each photoreceptor in a field to the nearest photoreceptor of the same type was determined for all photoreceptors in a given field. In order to avoid edge effects, only photoreceptors inside a 10 µm buffer zone around the perimeter of the field were analyzed. In order to generate spatial autocorrelograms each point in a photoreceptor distribution was placed at the origin of a coordinate system and then all other points were replotted relative to it. This procedure was repeated for every point in a given field. Density recovery profiles (DRPs) were derived from autocorrelograms by calculating the density of points within successive annuli out from the origin of the coordinate system. An annulus width of 0.3 microns was used, and the density was calculated by dividing the number of points within an annulus by the area of that annulus. The effective radius is a measure of the distance around a cell that is relatively devoid of other cells of the same type [41]. It is equal to the length of the base of a rectangle whose height is equal to the average cell density and which encloses an area equivalent to that produced by the dip in the DRP between the origin and the first abscissa where the DRP reaches the average cell density [26], [41], [72].

### Simulated Photoreceptor Distributions for Cross-Correlation Analysis

In order to create simulated distributions of points with a defined nearest neighbor regularity index for purposes of the cross-correlation analysis (Figure 5B), a sequential addition, hard disk model was used [51]. In this model random points are added sequentially to a field such that they cannot be placed within a defined distance of any previously placed points. This defined distance represents the hard disk diameter. Points are sequentially added until a desired density is attained. Increasing the hard disk diameter results in progressively more regular mosaics. In order to determine what hard disk diameter would yield simulated distributions with nearest neighbor regularity indices of the desired value, a series of simulations were conducted with a range of hard disk diameter and the resultant regularity indices were calculated (data not shown). A curve was then fitted to these datapoints, and the hard disk diameter necessary to achieve a given regularity index was read off of this curve.

As a control for the cross-correlation analysis, the regularity indices for heterotypic pairs ‘X-Y’ were calculated by using the coordinates of the real ‘X’ cells and comparing them to the simulated ‘Y’ mosaics. An additional feature of the simulated ‘Y’ mosaics was that they were created on a field already containing the real ‘X’ cells. Thus, newly placed ‘Y’ cells not only had to be at least one hard disk diameter from every previously placed ‘Y’ cell, but they also had to not overlap any ‘X’ cells. For the purpose of this simulation, cell diameter was assumed to be equal to oil droplet diameter (see Figure 1A). The average oil droplet diameters for all five oil droplet types were obtained by measuring ten different oil droplets of each type in a single field using an optical micrometer (data not shown). The mean oil droplet diameter shown in Figure 5A represents the average diameter for all five cone types combined.

## Supporting Information

Figure S1 Spatial distributions, autocorrelograms and density recovery profiles for all five cone types. (A–O) This figure depicts data in the same format as in Figure 3A–C for all five cone types in a single retinal field (dorsal-nasal field 7 in Table S1): double cones (A–C; included here for comparison), green cones (D–F), red cones (G–I), blue cones (J–L) and violet cones (M–O). The vertical orange lines in C, F, I, L and O indicate the average diameter of the oil droplet corresponding to each of the given cone types.
(2.21 MB TIF)

Figure S2 Cone photoreceptor mosaics with P_6_>∼0.47 obey Lemaître's law. (A and B) These two graphs depict the same data as in Figure 4F, split into two separate graphs with those datapoints having P_6_<∼0.47 in (A) and those with P_6_> = ∼0.47 in (B). The best fit power curve for both datasets are shown as dotted lines, and the equations are given in the box. The R-squared value for the goodness of fit to these curves is also shown. The solid line in both figures represents Lemaître's law. The value of the coefficient ([2π]^−1^) is shown numerically for comparison with the equation of the fit curve. The cone mosaics with P_6_>∼0.47 fit a curve which is almost directly superimposed on that representing Lemaître's law. In contrast, the cone mosaics with P_6_<∼0.47 show a relatively poor agreement with Lemaître's law.
(5.75 MB TIF)

Figure S3 Determining the global regularity indices for all four bird species. (A–F) Graphs of photoreceptor density vs. the inverse-square of the average nearest neighbor distance for the following datasets: computer-generated random mosaics (A), chicken cone mosaics (B), computer-generated perfect mosaics (C), *P. pubescens* cone mosaics (D), *P. domesticus* cone mosaics (E) and *C. livia* cone mosaics (F). Also shown are the best fit lines of the form, y  =  mx, for each dataset. Global regularity indices are equal to the inverse of the slope of the best fit line as shown, normalized to perfect which was set equal to one.
(5.15 MB TIF)

Table S1 Data and coordinates for cone mosaics from all four species. This file contains a total of 35 worksheets. ‘Summary’ includes a variety of data about all the P15 chicken mosaics (NND, nearest neighbor distance). Worksheets labeled ‘DN1’ (‘Dorsal-Nasal field #1’), ‘DT1’ (‘Dorsal-Temporal field #1’), ‘VN1’ (‘Ventral-Nasal field #1’), ‘NT1’ (‘Ventral-Temporal field #1’) etc. contain the raw coordinates for all P15 chicken fields examined in the present study. Worksheets labeled ‘E18’, ‘P0’ and ‘P6’ contain the raw coordinates for the chicken mosaics examined at the indicated developmental stages. Worksheets labeled ‘P. pubescens’, ‘P. domesticus’ and ‘C. livia’ contain the raw coordinates for the three additional species examined.
(6.86 MB XLS)
