# Supplementary figures and images for: Avian Cone Photoreceptors Tile the Retina as Five Independent, Self-Organizing Mosaics
Source: PLoS One. 2010 Feb 1;5(2):e8992. doi: 10.1371/journal.pone.0008992 (PMC2813877; doi:10.1371/journal.pone.0008992)

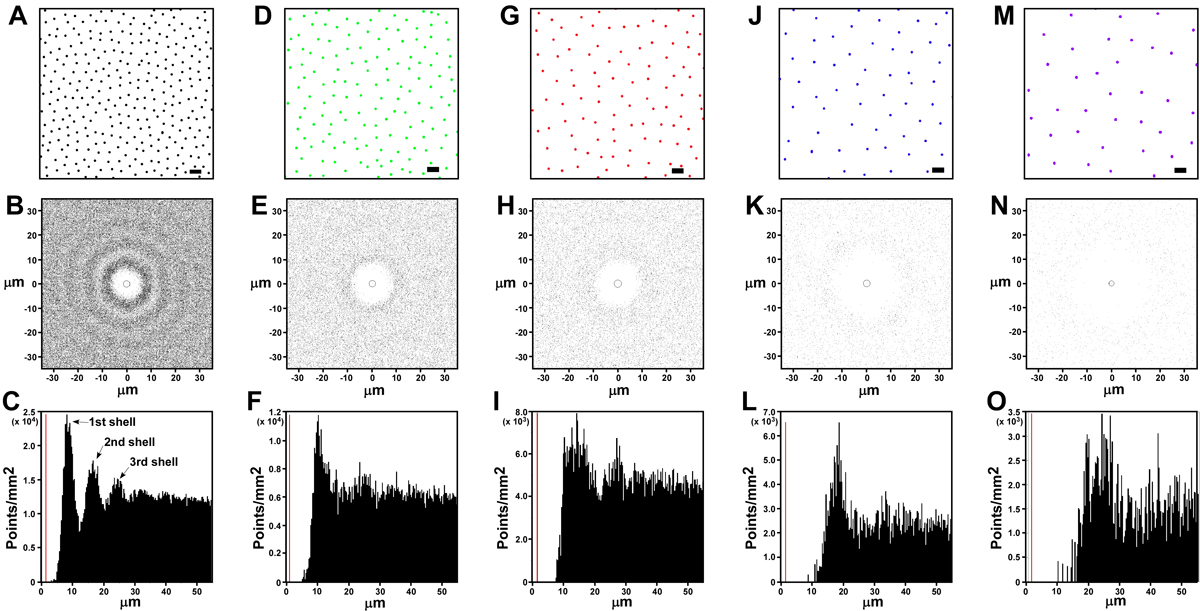

Supplement: Figure S1 — Spatial distributions, autocorrelograms and density recovery profiles for all five cone types. (A–O) This figure depicts data in the same format as in Figure 3A–C for all five cone types in a single retinal field (dorsal-nasal field 7 in Table S1): double cones (A–C; included here for comparison), green cones (D–F), red cones (G–I), blue cones (J–L) and violet cones (M–O). The vertical orange lines in C, F, I, L and O indicate the average diameter of the oil droplet corresponding to each of the given cone types. (2.21 MB TIF) [file pone.0008992.s001.tif]

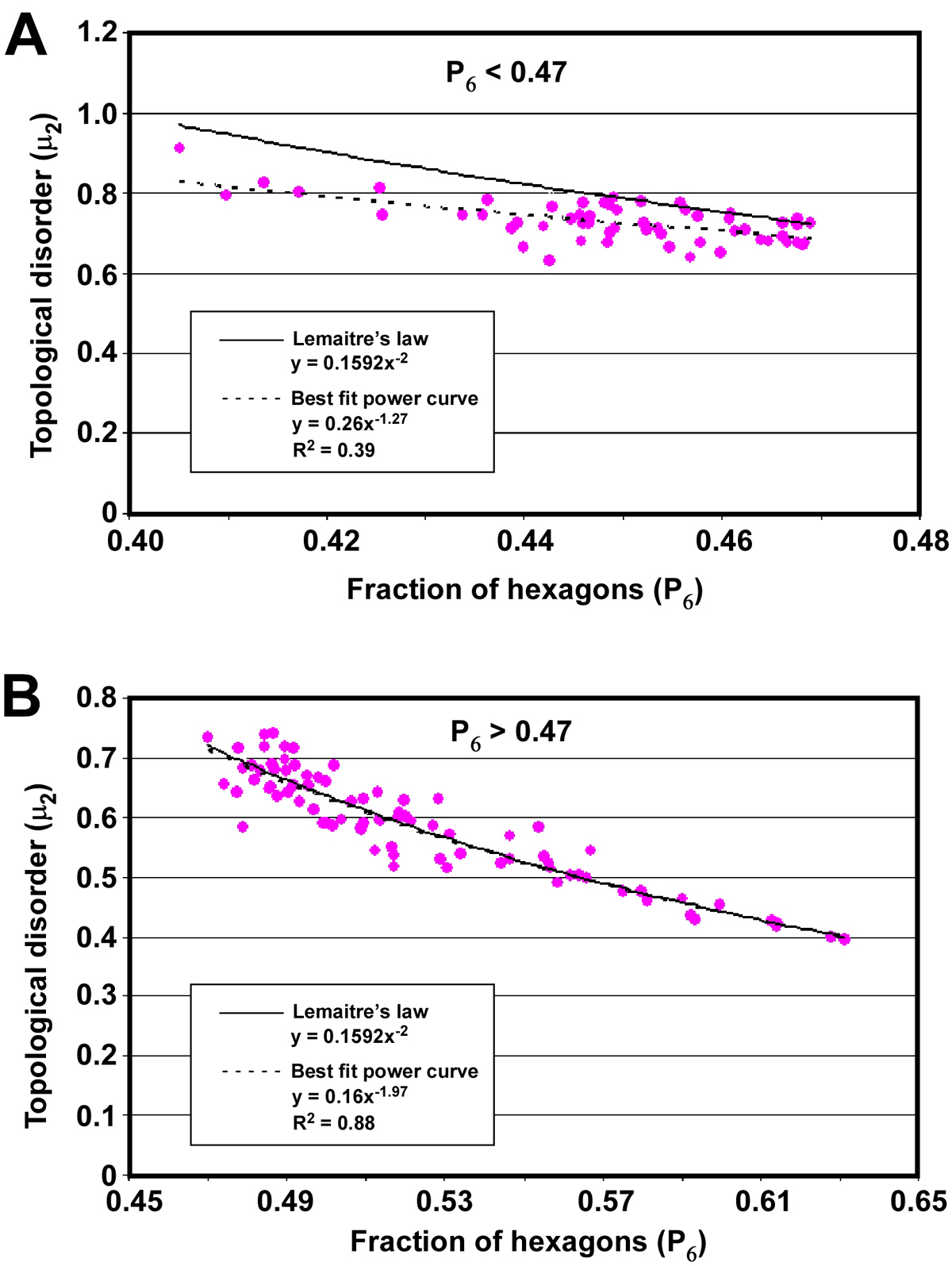

Supplement: Figure S2 — Cone photoreceptor mosaics with P6>∼0.47 obey Lemaître's law. (A and B) These two graphs depict the same data as in Figure 4F, split into two separate graphs with those datapoints having P6<∼0.47 in (A) and those with P6> = ∼0.47 in (B). The best fit power curve for both datasets are shown as dotted lines, and the equations are given in the box. The R-squared value for the goodness of fit to these curves is also shown. The solid line in both figures represents Lemaître's law. The value of the coefficient ([2π]−1) is shown numerically for comparison with the equation of the fit curve. The cone mosaics with P6>∼0.47 fit a curve which is almost directly superimposed on that representing Lemaître's law. In contrast, the cone mosaics with P6<∼0.47 show a relatively poor agreement with Lemaître's law. (5.75 MB TIF) [file pone.0008992.s002.tif]

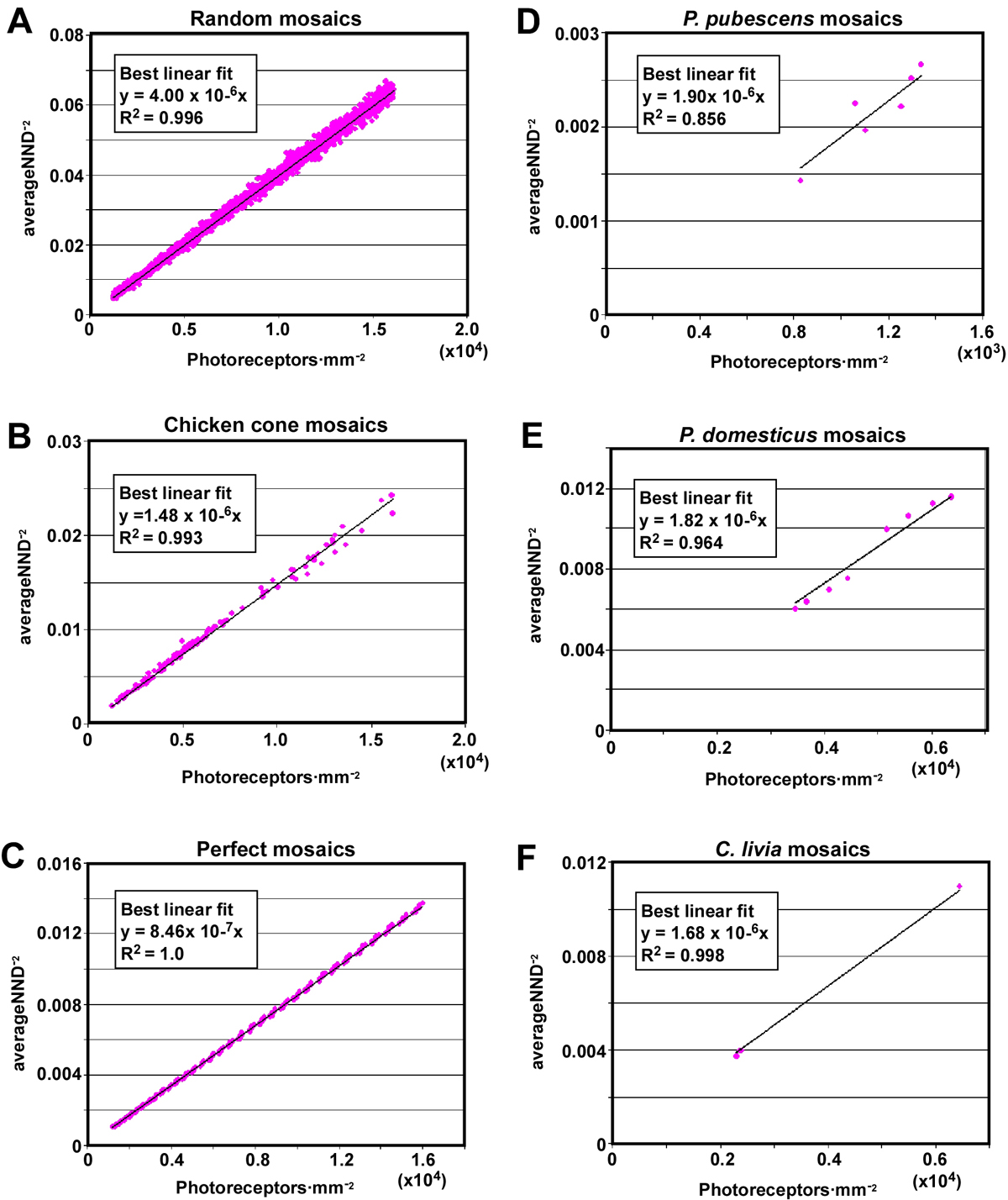

Supplement: Figure S3 — Determining the global regularity indices for all four bird species. (A–F) Graphs of photoreceptor density vs. the inverse-square of the average nearest neighbor distance for the following datasets: computer-generated random mosaics (A), chicken cone mosaics (B), computer-generated perfect mosaics (C), P. pubescens cone mosaics (D), P. domesticus cone mosaics (E) and C. livia cone mosaics (F). Also shown are the best fit lines of the form, y = mx, for each dataset. Global regularity indices are equal to the inverse of the slope of the best fit line as shown, normalized to perfect which was set equal to one. (5.15 MB TIF) [file pone.0008992.s003.tif]
